# PReS-FINAL-2045: Mutational analysis of sialic acid acetylesterase (siae) in juvenile idiopathic arthritis (JIA)

**DOI:** 10.1186/1546-0096-11-S2-P58

**Published:** 2013-12-05

**Authors:** E Tsitsami, E Sevdali, M Speletas

**Affiliations:** 1 1st Department of Pediatrics, School of Medicine, University of Athens, Children's Hospital "Aghia Sophia", Athens, Greece; 2 Department of Immunology & Histocompatibility, School of Medicine, University of Thessaly, Larissa, Greece

## Introduction

SIAE is involved in the maintenance of immunological tolerance through negative regulation of Β-cell receptor (BCR) signaling. Recent evidences, though conflicting, indicate that rare loss-of-function *SIAE *variants are associated with susceptibility to various autoimmune diseases. Advances in understanding JIA pathophysiology have led to the consensus that systemic JIA (SJIA) is an autoinflammatory disorder while oligo/polyarticular JIA (O/PJIA) is an antigen-driven lymphocyte-mediated autoimmune disease.

## Objectives

To elucidate whether *SIAE *variants predispose their carriers to O/PJIA but not to SJIA.

## Methods

Sixty-five JIA patients (M/F: 19/46, mean age: 9.8 years, range:2.5-18.3; 57 with O/PJIA and 8 with SJIA) and 82 age- and sex-matched healthy controls were enrolled. Amplification of all 10 *SIAE *exons, including exon-intron boundaries, and sequencing of purified products were performed.

## Results

Two novel heterozygous *SIAE *mutations, namely the Q343P (g.41498 A > C, c.1028A > C) and the Y495X (g.44266C > A, c.1485C > A), as well as three already described heterozygous *SIAE *mutations, namely the functionally innocent M89V (g.20536A > G) mutation and the silent mutations S156S (g.26573T > C) and T484T (g.44233G > A) were found in O/PJIA patients. The girl carrying the Q343P mutation had ANA(+) persistent oligoarthritis. Her family study proved that her father, having a family history of autoimmune disease, was also carrier of the same mutation. The girl with the Y495X mutation suffered from RF(-), ANA(+) polyarthritis. The novel *SIAE *mutations did not detected among normal controls. Amongst the patients with SJIA, one was heterozygote for the known functionally innocent K71R (g.11927A > G) and A467V (g.44181C > T) mutations as well as for the silent mutations T484T and S156S, while another one was heterozygote for the silent mutation R340R (g. 41490 T > C).

## Conclusion

Our results support the notion that SIAE might be involved to the pathogenesis of O/PJIA but not of SJIA. Functional analysis of the identified novel *SIAE *variants is required to prove the biological significance of these genetic alterations.

## Disclosure of interest

None declared.

